# Tomography patterns of lung disease in systemic sclerosis*

**DOI:** 10.1590/0100-3984.2015.0116

**Published:** 2016

**Authors:** Andréa de Lima Bastos, Ricardo de Amorim Corrêa, Gilda Aparecida Ferreira

**Affiliations:** 1 Adjunct Professor in the Department of Anatomy and Imaging at the Faculdade de Medicina da Universidade Federal de Minas Gerais (UFMG), Belo Horizonte, MG, Brazil.; 2 Associate Professor in the Department of Clinical Medicine at the Faculdade de Medicina da Universidade Federal de Minas Gerais (UFMG), Belo Horizonte, MG, Brazil.; 3 Adjunct Professor in the Department of Locomotor Studies at the Faculdade de Medicina da Universidade Federal de Minas Gerais (UFMG), Belo Horizonte, MG, Brazil.

**Keywords:** Sclerosis, Scleroderma, systemic, Radiology, Tomography, X-ray computed, Lung diseases

## Abstract

Currently, lung impairment is the leading factor responsible for the morbidity and mortality associated with systemic sclerosis. Therefore, the recognition of the various tomography patterns becomes decisive in the clinical management of these patients. In high-resolution computed tomography studies, the most common pattern is that of nonspecific interstitial pneumonia. However, there are other forms of lung involvement that must also be recognized. The aim of this study was to review the literature on the main changes resulting from pulmonary involvement in systemic sclerosis and the corresponding radiological findings, considering the current classification of interstitial diseases. We searched the Medline (PubMed), Lilacs, and SciELO databases in order to select articles related to pulmonary changes in systemic sclerosis and published in English between 2000 and 2015. The pulmonary changes seen on computed tomography in systemic sclerosis are varied and are divided into three main categories: interstitial, alveolar, and vascular. Interstitial changes constitute the most common type of pulmonary involvement in systemic sclerosis. However, alveolar and vascular manifestations must also be recognized and considered in the presence of atypical clinical presentations and inadequate treatment responses.

## Abstract (pt)

O comprometimento pulmonar pela esclerose sistêmica é, atualmente, o maior responsável pela morbidade e mortalidade relacionadas à doença e, assim, o reconhecimento dos seus vários padrões de alterações tomográficas se torna decisivo na abordagem destes pacientes. Embora a apresentação tomográfica mais comum, em estudos de alta resolução, seja a pneumonia intersticial não específica, existem outros achados clínicos e de imagem que não correspondem a esse padrão. O objetivo deste estudo foi realizar uma revisão da literatura sobre as principais alterações pulmonares decorrentes do comprometimento pela esclerose sistêmica e de suas manifestações radiológicas, considerando-se a classificação atual das doenças intersticiais. Foi realizada uma seleção de estudos das alterações pulmonares na esclerose sistêmica entre os anos de 2000 e 2015, por meio de mecanismos de busca em publicações de língua inglesa, na base de dados Medline/PubMed, Lilacs e SciELO. As alterações tomográficas pulmonares encontradas na esclerose sistêmica têm padrão variado e podem ser agrupadas em três principais categorias: alterações intersticiais, alveolares e vasculares. As alterações intersticiais são as mais frequentemente relacionadas à doença pulmonar na esclerose sistêmica, mas as manifestações alveolares e vasculares devem ser reconhecidas e consideradas na presença de apresentações clínicas atípicas e resposta inadequada à terapia instituída.

## INTRODUCTION

Systemic sclerosis (SSc) is an autoimmune connective tissue disease, of unknown cause, characterized by inflammatory changes, fibrosis, obstructive small-vessel vasculopathy, and collagen deposition in various organs, especially the lungs^(1,2)^. The disease is more common in women than in men, at an average ratio ranging from 3:1 to 8:1, and its incidence peaks between 45 and 64 years of age^(3,4)^.

Pulmonary involvement is a major feature of the evolution of SSc, the incidence of such involvement ranging from 70% to 90%, and is currently the leading cause of morbidity and mortality among individuals with SSc^(4)^.

In SSc patients, inflammation and interstitial fibrosis are seen in SSc patients, as are excessive deposition of extracellular matrix and vascular obliteration, which can also cause pulmonary hypertension^(5)^. The resulting anatomical changes have variable radiological manifestations. The early detection and proper interpretation of the radiological findings constitute a critical step in deciding when is the proper time to start treatment.

The pulmonary aspect of the disease has a clinical course that can range from presentations that are more indolent to those that progress rapidly, with a corresponding rapid decline in lung function. In some cases, the pulmonary fibrosis precedes the appearance of systemic disease by a number of years. Respiratory symptoms, present in more than 50% of patients with SSc, do not constitute reliable indicators of impairment of the lung parenchyma, because they can also arise from the pulmonary vascular impairment or muscle weakness associated with the disease^(2,6)^.

The pulmonary changes arising from SSc can be demonstrated by diagnostic imaging methods. Such methods include high-resolution computed tomography (HRCT) of the chest, which allows the anatomy of the lung parenchyma to be detailed with a sensitivity far superior to that of X-ray, constituting an important tool for the diagnosis and evaluation of the extent of the disease^(7)^.

The aim of this study was to review the literature on the main pulmonary changes resulting from SSc and the corresponding radiological manifestations.

## METHODS

We selected articles by conducting searches in the Medline (PubMed), Lilacs, and SciELO databases, using the following keywords: scleroderma; systemic sclerosis, radiology, computed tomography, and lung diseases. We further limited our searches to articles published in English and involving human subjects. Considering the approaches taken by the authors of studies employing HRCT and, primarily, changes in the consensus for interstitial lung disease, we selected 21 articles, published between 2000 and 2015, that included descriptive information related to radiological aspects of lung changes secondary to SSc. To identify additional relevant references, we performed hand searches of the bibliographies of the selected articles.

## DISCUSSION

### Interstitial changes

There are various forms of pulmonary involvement in SSc, interstitial disease being the most common. Interstitial changes in SSc ares characterized by diffuse pulmonary distribution and can correspond to the various histopathological subtypes. The use of HRCT plays an important role in the topographic identification of SSc lesions, as well as in evaluating their extent and monitoring the evolution of the process.

Initial descriptions of the pathological features of the disease come from post-mortem studies, which have suggested a pattern of fibrosis with little inflammatory response, although the presence of inflammation and fibrosis in the alveolar walls has recently been discussed^(8)^. In one autopsy study, fibrosis was observed in more than 75% of the cases^(9)^.

Fibrosis caused by SSc is the most frequently observed histological component, with a variable clinical evolution^(10)^. Of all cases of pulmonary fibrosis in SSc, 50.0-77.5% are due to nonspecific interstitial pneumonia, which could explain that difference in prognosis^(10,11)^. Nonspecific interstitial pneumonia is a particular form of idiopathic interstitial pneumonia characterized by varying degrees of inflammation and fibrosis, from forms predominated by inflammatory processes to those predominated by fibrosis, which are the most common, although without the fibroblastic foci and honeycombing seen in usual interstitial pneumonia^(8,12)^.

**Figure 1:**
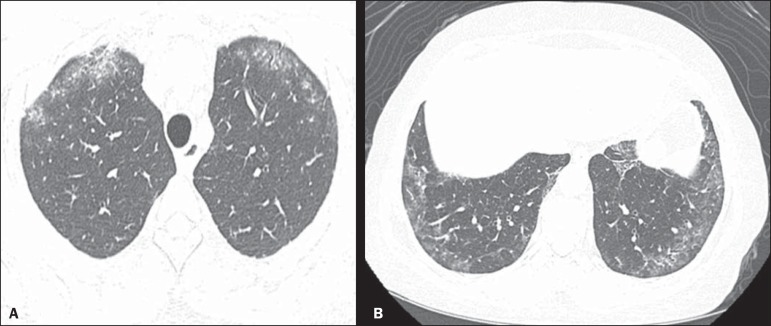
A 55-year-old female with an 8-year history of SSc, without respiratory symptoms. Note the bilateral areas of ground-glass attenuation in the cortical regions of the upper lobes (**A**) and of the basal segments of the lower lobes (**B**).

**Figure 2:**
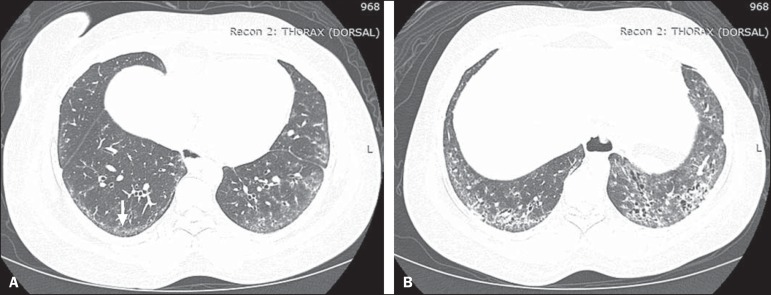
A 37-year-old female with a 10-year history of SSc, presenting with severe dyspnea and esophageal dilation. HRCT showing, in **A**, ground-glass attenuation, sparing the subpleural regions (arrow), and, in **B**, traction bronchiectasis, thickening of the interlobular septa, and subpleural lines, accompanied by ground-glass attenuation with a cortical distribution.

The abnormalities seen in nonspecific interstitial pneumonia, most often observed in HRCT, include irregular, reticular, ground-glass attenuation, as well as traction bronchiectasis and bronchiolectasis (Figure 1). These lesions rarely occur in the subpleural regions of the lungs (Figure 2), and that aspect can be useful in differentiating nonspecific interstitial pneumonia from usual interstitial pneumonia^(12)^.

**Figure 3:**
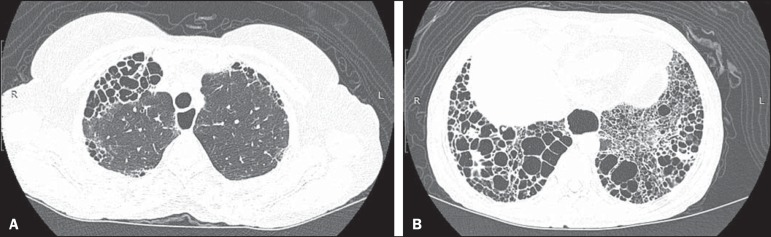
A 58-year-old female with a 26-year history of SSc, presenting with cough, pronounced dyspnea, and bronchospasm. HRCT showing significant esophageal dilation and extensive bilateral honeycombing, predominantly in the upper lobes (**A**) and lower lobes (**B**), with a pattern characteristic of usual interstitial pneumonia.

Usual interstitial pneumonia is a less common presentation in SSc. The typical HRCT pattern in usual interstitial pneumonia is characterized by reticular changes, predominantly in the basal, peripheral, and subpleural regions, together with honeycombing, traction bronchiectasis, traction bronchiolectasis, architectural distortion, and volume loss in the affected region^(13)^. This pattern has a worse prognosis than does that observed in nonspecific interstitial pneumonia (Figure 3).

### Alveolar changes

Less common forms of pulmonary involvement reported in SSc are diffuse alveolar damage, pulmonary hemorrhage, organizing pneumonia, aspiration pneumonia, and lung disease associated with the drugs used in the treatment^(6)^.

There have been only a few reported cases of organizing pneumonia as a manifestation of SSc. However, because the gold standard for diagnosis is open-lung biopsy, which is rarely performed in these patients, the true incidence of organizing pneumonia in SSc is unknown. Taylor et al.^(14)^reported three cases of organizing pneumonia diagnosed by open-lung biopsy in patients with SSc.

**Figure 4:**
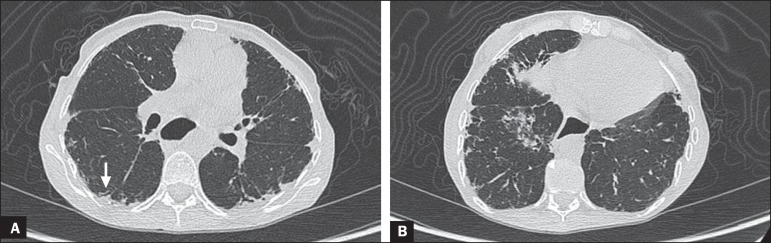
A 49-year-old female with a 10-year history of SSc, without respiratory symptoms. HRCT with a lung window, showing small subpleural focal consolidations interspersed with bronchiectasis (**A**, arrow) in the middle and lower thirds of the lung segments (**A** and **B**, respectively), together with marked esophageal dilation. The radiological pattern is suggestive of organizing pneumonia, even before the histopathological study.

HRCT can reveal pronounced bilateral consolidation in the subpleural space (Figure 4) or in the peribronchial areas, with a migratory aspect and some areas of ground-glass attenuation. The reversed halo sign can be useful in suggesting the diagnosis. Histologically, organizing pneumonia involves the alveoli and alveolar ducts, with or without intraluminal bronchial polyps^(12)^. Clinically, the disease manifests as cough, fever, and dyspnea; there is a predominance of lymphocytes in the bronchoalveolar lavage fluid^(12,14)^. Other authors have reported this same presentation^(15-17)^.

In the case of atypical clinical and radiological presentations, open-lung biopsy should be considered, given that organizing pneumonia and fibrosing alveolitis differ in terms of prognosis and treatment. The former responds to corticosteroids, whereas the latter often requires the use of immunosuppressive agents^(14)^.

Because pulmonary changes that are detectable by routine chest X-ray in only 25-53% of cases can be detected with HRCT in up to 94% of cases, HRCT is the method of choice for the investigation of interstitial lung disease in SSc^(18)^.

In patients with SSc, aspiration pneumonia occurs due to esophageal involvement secondary to the disease. Esophageal involvement is seen in 50-90% of cases of SSc, symptoms arising from dysmotility and reflux^(19)^. On CT scans of SSc patients, the coronal diameter of the esophageal lumen is increased, ranging from 1.2 cm to 4.0 cm, with a mean of 2.3 cm^(20)^.

### Vascular changes

The pulmonary vascular involvement in SSc can cause pulmonary arterial hypertension, which results from the increase in pulmonary vascular resistance caused by occlusion and remodeling of the pulmonary arterioles.

**Figure 5:**
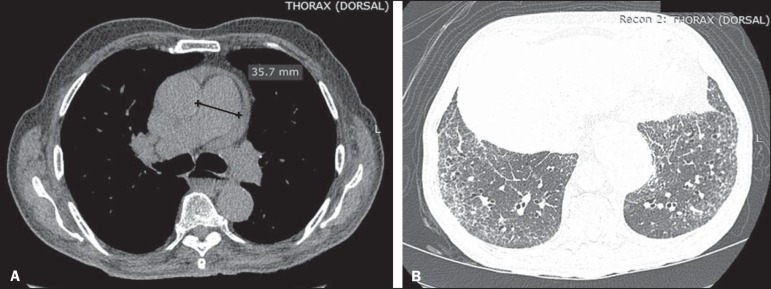
An 81-year-old male with a 10-year history of SSc. **A:** HRCT with a mediastinal window showing an increase in the caliber of the pulmonary artery trunk (35.7 mm) and esophageal dilation with an air-fluid level inside. **B:** Areas of ground-glass attenuation in the pulmonary parenchyma, with thickening of the interlobular septa, interspersed with traction bronchiectasis, predominantly cortical in distribution, suggestive of nonspecific interstitial pneumonia.

Pulmonary arterial hypertension, defined as a mean pulmonary artery pressure equal to or greater than 25 mmHg and a pulmonary artery occlusion pressure equal to or less than 15 mmHg, is a major cause of SSc-related morbidity and mortality (Figure 5), mean survival after diagnosis ranging from 1.5 to 3 years^(21)^.

This manifestation can occur in isolation or can be accompanied by interstitial lung disease, which worsens its prognosis. A diagnosis based on clinical symptoms alone is limited by the presence of other pulmonary changes that can manifest in a similar manner^(2,22)^.

On HRCT scans, increases in the caliber of the pulmonary artery trunk (normal value: 28.6 ± 2 mm) and main pulmonary arteries are often observed, although their absence does not exclude the diagnosis. A finding of pericardial effusion, especially in the anterior recess, with a thickness > 10 mm, is indicative of a poor prognosis and is also a strong predictor of pulmonary arterial hypertension on echocardiography^(23-26)^.

Another pulmonary vascular manifestation in SSc is veno-occlusive disease, which is characterized by intimal proliferation and fibrosis of the intrapulmonary veins and venules, together with arteriolar involvement. The definitive diagnosis is obtained by biopsy, and alternatively can be performed clinically, in view of the increased risk of post-operative complications^(22)^. Recent histological studies have shown that the venous involvement in the pulmonary form of SSc is greater than previously described, which explains, at least in part, the fact that patients with SSc-related pulmonary arterial hypertension are more likely to be refractory to treatment than are those with the idiopathic form^(27)^. In patients with SSc-related pulmonary arterial hypertension, HRCT reveals centrilobular ground-glass attenuation, septal thickening, and lymph node enlargement^(22)^.

### Other changes

Some of the changes observed on CT scans of patients with SSc can be concomitant to findings of parenchymal disease and merit attention because of their frequency or severity.

A common finding in patients with SSc, with a prevalence of 41-74%, is lymphadenopathy, which appears to be associated with the presence of chronic interstitial lung disease secondary to an inflammatory response to aspiration pneumonia and reflux or other concomitant diseases such as lymphoma and sarcoidosis^(24,28)^.

Some studies have shown that the incidence of neoplastic diseases, especially those involving the lungs, is a higher among individuals with SSc. The overall incidence of neoplasms in SSc patients has been reported to be as high as 10.7%, and the risk of developing lung cancer is up to seven times greater among smokers with SSc than among other smokers^(29)^.

## CONCLUSION

Pulmonary complications arising from SSc can have a quite adverse clinical course, and the main clinical dilemma is related to the indication for and appropriate timing of immunosuppression therapy. Immunosuppressive agents play an important role in slowing the progression of fibrosis, and potential adverse effects related to such medication should be considered, being essential to the recognition of various radiological patterns of pulmonary disease.

The changes most frequently observed in SSc are those that result from interstitial involvement, although manifestations related to alveolar and vascular involvement should always be borne in mind in cases in which the clinical picture is atypical and there is an inadequate response to the treatment, due to the need for an immediate, specific therapeutic approach.

Other thoracic manifestations of SSc are also observed on CT scans of the chest and can be associated with lung disease. Such changes should also be recognized and correlated with the clinical findings in order to inform the handling of cases.
